# Supplementary material for: Multi-Epitopic Peptide Vaccine Against Newcastle Disease Virus: Molecular Dynamics Simulation and Experimental Validation
Source: Vaccines (Basel). 2024 Nov 1;12(11):1250. doi: 10.3390/vaccines12111250 (PMC11598688; doi:10.3390/vaccines12111250)
Supplement: Supplementary file 1 [file vaccines-12-01250-s001.zip › vaccines-3267052-supplementary.pdf]

Table S1. NDV genomic isolated from Pakistan

**21**	**JQ517285**	**Chicken/Pakistan/NDV/UD28**	**Chicken**	**2011**
**22**	GU182323	Chicken/SPVC/Karachi/NDV/43	Chicken	2008
**23**	GU182325	Chicken/SPVC/Karachi/23	Chicken	2004
**24**	GU182323	SPVC/Karachi/NDV/43		2008
**25**	GU182323	Chicken//SPVC/Karachi/NDV/43	Chicken	2008
**26**	GU182329	Chicken/Karachi/NDV/26	Chicken	2005
**27**	JQ517285	Chicken/Pakistan/NDV/UDL8	Chicken	2011
**28**	JN682208	Chicken/B4P/Pakistan	Chicken	2010
**29**	JN682187	Chicken/B4P/Rawalpindi	Chicken	2010
**30**	JN682188	Chicken/CP/Attock	Chicken	2010
**31**	GU182331	SPVC/Karachi/NDV/33		2007
**32**	GU182329	Chicken/SPVC/Karachi/NDV/26	Chicken	2005
**33**	JN682191	Chicken/CP/Islamabad3	Chicken	2010
**34**	JX436343	Chicken/MM15/Okara	Chicken	
**35**	JX436340	Chicken/MM18/Faisalabad	Chicken	
**36**	JX436344	Chicken/MM14/Lahore	Chicken	
**37**	JX436342	Chicken/MM16/Gujranwala	Chicken	
**38**	JX436341	Chicken/MM15/Sheikhupura	Chicken	

Table S2: Physiochemical properties of vaccine construct

**Parameter**	**Score**
Number of amino acids	40
Molecular weight	4441.27
Theoretical pI	9.9
Estimated half-life	5.5 h (mammalian reticulocytes, in vitro)
Instability index (II)	33.71
Aliphatic index (AI)	124.25
*GRAVY score	-0.347
Antigenicity	0.7587

*** Grand average of hydropathicity**

Table S3: Statistical values of docking complex (TLR4 receptor and vaccine construct)

HADDOCK score	-67.0 +/- 1.9
Cluster size	200
RMSD from the overall lowest-energy structure	0.5 +/- 0.3
Van der Waals energy	-95.3 +/- 10.4
Electrostatic energy	-107.7 +/- 19.3
Desolvation energy	-40.2 +/- 4.2
Restraint's violation energy	900.8 +/- 99.2
Buried Surface Area	3095.0 +/- 77.1
Z-Score	0.0

Table S4: Statistical values of docking complex (MHC-I receptor and vaccine construct 1)

**HADDOCK score**	-73.3 +/- 2.1
**Cluster size**	200
**RMSD from the overall lowest-energy structure**	0.3 +/- 0.2
**Van der Waals energy**	-18.0 +/- 2.0
**Electrostatic energy**	-216.5 +/- 15.9
**Desolvation energy**	-39.2 +/- 2.3
**Restraint's violation energy**	272.9 +/- 39.8
**Buried Surface Area**	2990.5 +/- 51.4
**Z-Score**	0.0

```
                                  20                  40                  60
F consensus  MGSKPSTRIPVPLSLITRVILILSCICLTSSLDGRPLAAAGIVVTGDKAVNIYTSSQTGSIIVKLLPN
                          80                 100                 120
F consensus  MPKDKEACAKAPLEAYNRTLTTLLTPLGDSIRRIQGSVSTSGGRRQKRFIGAVIGSVALGVATAAQIT
                140                 160                 180                 200
F consensus  AAAALIQANQNAANILRLKESIAATNEAVHEVTDGLSQLAVAVGKMQQFVNDQFNNTARELDCIKITQ
                               220                 240                 260
F consensus  QVGVELNLYLTELTTVFGPQITSPALTQLTIQALYNLAGGNMDYLLTKLGVGNNQLSSLIGSGLIAGY
                     280                 300                 320                 340
F consensus  PILYDSQTQLLGIQVNLPSVGNLNNMRATYLETLSVSTTKGFASALVPKVVTQVGSVIEELDTSYCIE
                                  360                 380                 400
F consensus  SNLDLYCTRIVTFPMSPGIYSCLSGNTSACMYSKTEGALTTPYMTLKGSVIANCKITTCRCADPPGII
                          420                 440                 460
F consensus  SQNYGEAVSLIDRHSCNVLSLDGITLRLSGEFDATYQKNISILESQVIVTGNLDISTELGNVNNSISN
                480                 500                 520                 540
F consensus  ALDKLAESNSKLDKVNVRLTSTSALITYIVLTAVSLVSGALSLVLSCYLMYKQKAQQKTLLWLGNNTL

F consensus  DQMRATTRA
```

Figure S1. Concensus sequence of F NDV protein.

```
                                    20                  40                  60
HN consences  MSRAVNRVMLENEEREAKNTWRLVFRIAVLLLMIMILAISAAALAYSMGTSTPRDLTGISVAISKTE
                            80                 100                 120
HN consences  DKVTSLLSSSQDVMDRIYKQVALESPLALLNTESIIMNAITSLSYQINGAANNSGCGAPVHDPDYIG
                  140                 160                 180                 200
HN consences  GIGKELIVDDTSDVTSFYPSAYQEHLNFIPAPTTGSGCTRIPSFDMSTTHYCYTHNVILSGCRDHSH
                                 220                 240                 260
HN consences  SHQYLALGVLRTSATGKVFFSTLRSINLDDTQNRKSCSVSATPLGCDILCSKVTETEEEDYKSVTPT
                       280                 300                 320
HN consences  SMVHGRLGFDGQYHEKDLDTTALFKDWVANYPGVGGGSFVDERVWFPVYGGLKPNSPSDTAQEGKYV
                 340                 360                 380                 400
HN consences  IYKRYNDTCPDKQDYQIRMAKSSYKPGRFGGKRVQQAILSIKVSTSLGEDPMLTIPPNTITLMGAEG
                                420                 440                 460
HN consences  RILTVGTSHFLYQRGSSYFSPALLYPMTISNKTATLHSPYTFNAFTRPGSVPCQASARCPNSCITGV
                        480                 500                 520
HN consences  YTDPYPLIFHRNHTLRGVFGTMLDDGQARLNPVSAVFDDISRSRVTRVSSSSTKAAYTTSTCFKVVK
                 540                 560
HN consences  TNKTYCLSIAEISNTLFGEFRIVPLLVEILKDNRA
```

Figure S2. Concensus sequence of NDV HN protein.

(B)

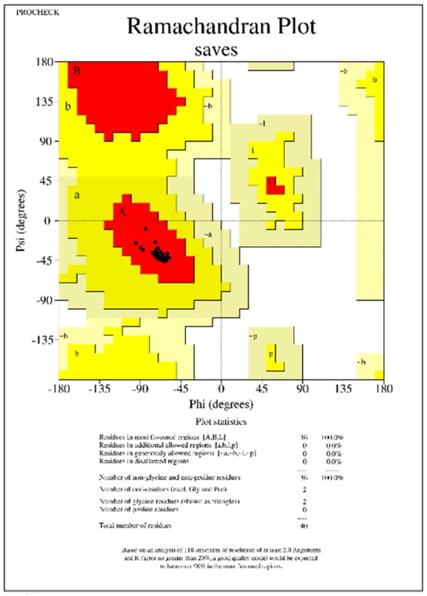

Figure S3. Ramachandran plot of modeled peptide.

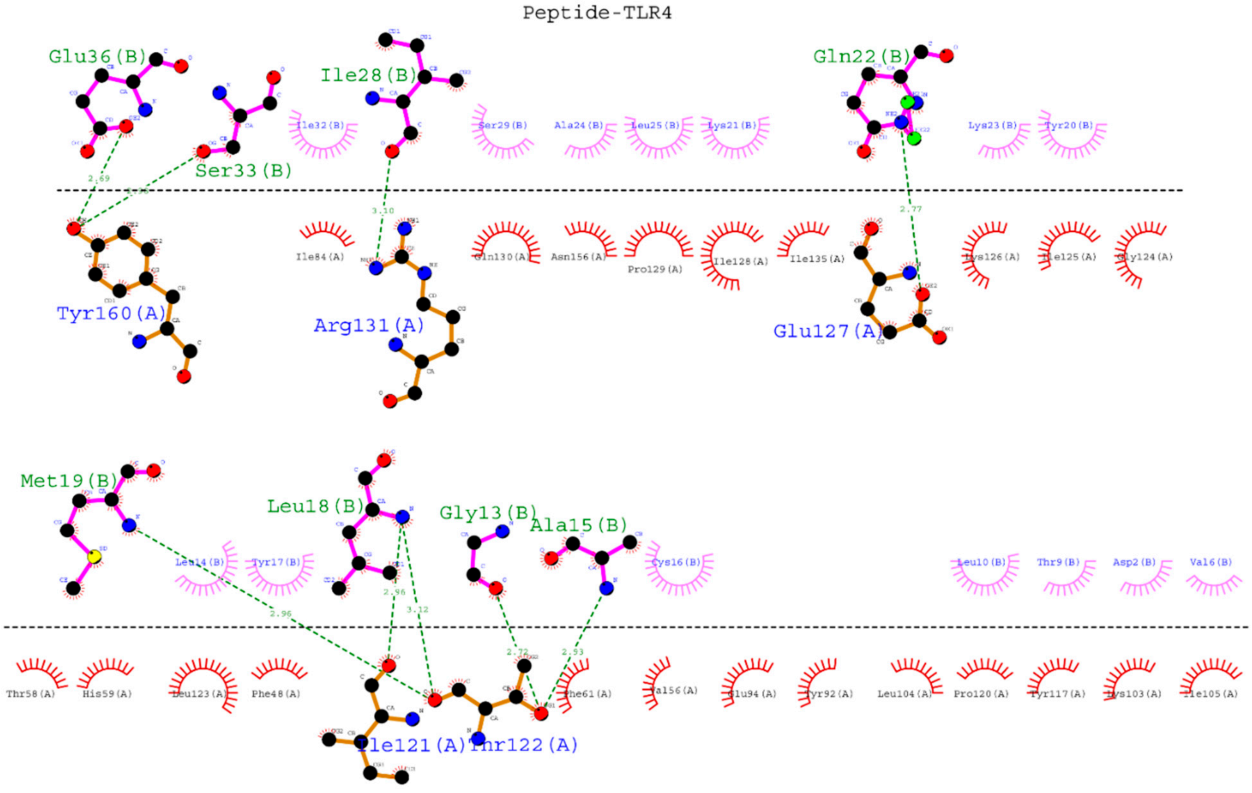

Figure S4. Peptide vaccine and TLR4 interactions.

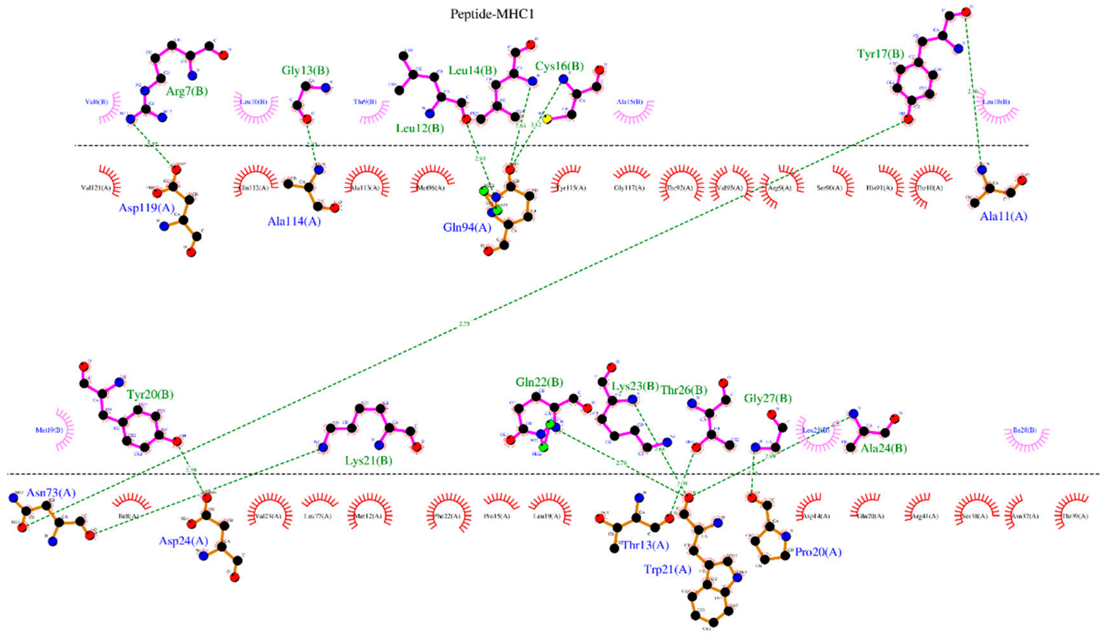

Figure S5. Peptide vaccine and MHC1 interactions.
